# A Recently Assembled Degradation Pathway for 2,3-Dichloronitrobenzene in *Diaphorobacter* sp. Strain JS3051

**DOI:** 10.1128/mBio.02231-21

**Published:** 2021-08-24

**Authors:** Tao Li, Yi-Zhou Gao, Jia Xu, Shu-Ting Zhang, Yuan Guo, Jim C. Spain, Ning-Yi Zhou

**Affiliations:** a State Key Laboratory of Microbial Metabolism, Joint International Research Laboratory of Metabolic and Developmental Sciences, and School of Life Sciences and Biotechnology, Shanghai Jiao Tong University, Shanghai, China; b Center for Environmental Diagnostics & Bioremediation, University of West Florida, Pensacola, Florida, USA; University of Washington

**Keywords:** 2,3-dichloronitrobenzene, chlorocatechol 1,2-dioxygenase, evolution, Nag-like dioxygenase, nitroarene, nitroaromatic

## Abstract

*Diaphorobacter* sp. strain JS3051 utilizes 2,3-dichloronitrobenzene (23DCNB), a toxic anthropogenic compound, as the sole carbon, nitrogen, and energy source for growth, but the metabolic pathway and its origins are unknown. Here, we establish that a gene cluster (*dcb*), encoding a Nag-like dioxygenase, is responsible for the initial oxidation of the 23DCNB molecule. The 2,3-dichloronitrobenzene dioxygenase system (DcbAaAbAcAd) catalyzes conversion of 23DCNB to 3,4-dichlorocatechol (34DCC). Site-directed mutagenesis studies indicated that residue 204 of DcbAc is crucial for the substrate specificity of 23DCNB dioxygenase. The presence of glutamic acid at position 204 of 23DCNB dioxygenase is unique among Nag-like dioxygenases. Genetic, biochemical, and structural evidence indicate that the 23DCNB dioxygenase is more closely related to 2-nitrotoluene dioxygenase from *Acidovorax* sp. strain JS42 than to the 34DCNB dioxygenase from *Diaphorobacter* sp. strain JS3050, which was isolated from the same site as strain JS3051. A gene cluster (*dcc*) encoding the enzymes for 34DCC catabolism, homologous to a *clc* operon in Pseudomonas knackmussii strain B13, is also on the chromosome at a distance of 2.5 Mb from the *dcb* genes. Heterologously expressed DccA catalyzed ring cleavage of 34DCC with high affinity and catalytic efficiency. This work not only establishes the molecular mechanism for 23DCNB mineralization, but also enhances the understanding of the recent evolution of the catabolic pathways for nitroarenes.

## INTRODUCTION

Synthetic nitroarenes, such as nitrobenzene (NB), nitrotoluenes (NTs), and chloronitrobenzenes (CNBs), have been widely used as chemical synthesis feedstocks in the production of pesticides, pharmaceuticals, and dyes (1). These chemicals have been introduced into the environment due to anthropogenic activities and many of them have caused serious contamination (2–4). Evolution of bacterial catabolic pathways in response to selection by the presence of synthetic nitroarenes has been established by the isolation of strains capable of growing on them, including the nitrobenzene utilizer *Comamonas* sp. strain JS765 (5), 2NT utilizer *Acidovorax* sp. strain JS42 (6), 2,4-dinitrotoluene utilizers Burkholderia cepacia R34 (7) and *Burkholderia* sp. strain DNT (8), 3,4-dichloronitrobenzene (34DCNB) utilizer *Diaphorobacter* sp. strain JS3050 (9), 2CNB utilizer Pseudomonas sp. strain ZWLR2-1 (10), and 4CNB utilizer *Comamonas* sp strain CNB-1 (11). Insights about the catabolic mechanisms of nitroarene biodegradation and evolutionary origins of the pathways are emerging (12–15). The limited history of these synthetic nitroaromatic compounds in the biosphere makes it reasonable to assume that evolution of the catabolic pathways was through recruitment and evolution of existing genes related to the degradation of natural organic compounds (16, 17). The oxidative biodegradation pathways of nitroarenes have been regarded as a model for the recruitment and assembly of metabolic pathways in response to novel chemicals (13).

A common mechanism of initial attack on nitroarenes leading to productive catabolic pathways is catalyzed by the ring-hydroxylating dioxygenases that belong to the Rieske non-heme iron oxygenase family (14). The enzyme system comprises an oxidoreductase, an iron-sulfur ferredoxin protein, and a terminal oxygenase center (α-subunit and β-subunit). All of the nitroarene dioxygenases identified so far share an ancestor with naphthalene dioxygenase (Nag) from strains such as *Ralstonia* sp. U2 (18), with the exception of 3NT dioxygenase (a biphenyl-like dioxygenase) from *Rhodococcus* sp. strain ZWL3NT (19). The lower catabolic pathways involved in oxidative degradation of nitroarenes vary considerably depending on the nature and location of the additional substituent groups. For example, genes encoding chlorocatechol degradation were recruited for assembly of a catabolic pathway for chloronitrobenzene (20, 21). In contrast, genes encoding degradation of methylnitrocatechols were recruited for degradation of 2,4-DNT (7, 18).

A limited number of bacterial strains are known to use CNBs as the sole carbon and nitrogen source for growth. Among them, *Comamonas* sp. strain LW1 (22), Pseudomonas putida ZWL73 (23), and *Comamonas* sp. strain CNB-1 (11) can degrade 4-chloronitrobenzene (4CNB) through a partially reductive pathway. Pseudomonas stutzeri ZWLR2-1 degrades 2-chloronitrobenzene (2CNB) via an oxidative pathway (10). A multicomponent dioxygenase catalyzes the dioxygenation of 2CNB to form nitrite and 3-chlorocatechol, which is subsequently degraded via the *ortho* ring-cleavage pathway (20). Engineered strains that could grow on all three CNBs isomers were generated by combination of the NBDO or its variants with the chlorocatechol pathway (24), which reflects the plasticity of the system.

In contrast, biodegradation of dichloronitrobenzenes (DCNBs) was reported only recently (9). DCNBs are primarily used as precursors to dichloroanilines, which are widely used in synthesis of pesticides, dyes, and herbicides. The United States Environmental Protection Agency (EPA) has included 23DCNB in its list of high production volume chemicals (greater than one million pounds per year) (25). Contaminating 23DCNB can enter the environment at manufacturing sites (9) and has been detected in industrial wastewater, drinking water, and fish samples (26); 23DCNB is a severe skin irritant and is genotoxic (26, 27).

*Diaphorobacter* sp. strains JS3050 and JS3051, isolated from the same DCNB-contaminated site, could utilize 34DCNB and 23DCNB, respectively, as the sole sources of carbon, nitrogen, and energy (9). Preliminary evidence indicated that both strains metabolized DCNBs via oxidative rather than reductive pathways, based on nitrite release. The recently characterized metabolic pathway of 34DCNB in strain JS3050 involves initial conversion of 34DCNB into 45DCC catalyzed by a dioxygenase (DcnAaAbAcAd) that is closely related to 2,4-DNT dioxygenase. The resultant 45DCC is subjected to ring-cleavage by DcnC, which shows 95% identity to a broad-substrate-spectrum chlorocatechol 1,2-dioxygenase (TetC) from Pseudomonas chlororaphis RW71 (21). It is unknown whether the 23DCNB catabolic pathway and its genetic determinants are similar to those of 34DCNB. In addition, these two strains provide an opportunity to study how pathways for the closely related isomers evolved in the same microbial community exposed to both DCNB isomers.

In this study, we elucidated the 23DCNB catabolic pathway in strain JS3051 via genome sequencing, whole-cell biotransformations, recombinant expression, and biochemical analyses. Comparative genome analysis, substrate specificity, site-directed mutagenesis, and structural analysis revealed the origins of the pathway and the factors that dictate the different recent origins of the 23DCNB and 34DCNB dioxygenases.

## RESULTS

### Genome of *Diaphorobacter* sp. strain JS3051.

The complete genome of strain JS3051 comprises 4.6 Mb, consisting of one circular chromosome and three circular plasmids. More details of the genomic information are summarized in Table S1 in the supplemental material. The two identical 16S rRNA gene sequences of strain JS3051 share 100% identity to those of *Acidovorax* sp. strain JS42, Acidovorax ebreus strain TPSY, and Diaphorobacter polyhydroxybutyrativorans strain SL-205, and 99.93% identity (1 nucleotide difference) to that of *Diaphorobacter* sp. strain JS3050. Comparison of the whole genomes of the above five strains revealed that strain JS3051 had the highest identity to *Acidovorax* sp. strain JS42 and *Diaphorobacter* sp. strain JS3050 (Fig. S1). The closest relationship was further identified between strain JS3051 and *Acidovorax* sp. strain JS42 by calculating the distances between species derived from *in silico* DDH (with a 0.81 DNA-DNA hybridization [DDH]) (Table S2). The probability of DDH > 0.7 was 96%, which reaches the threshold value of same species (28).

### Prediction and organization of 23DCNB catabolic genes.

**FIG 1:**
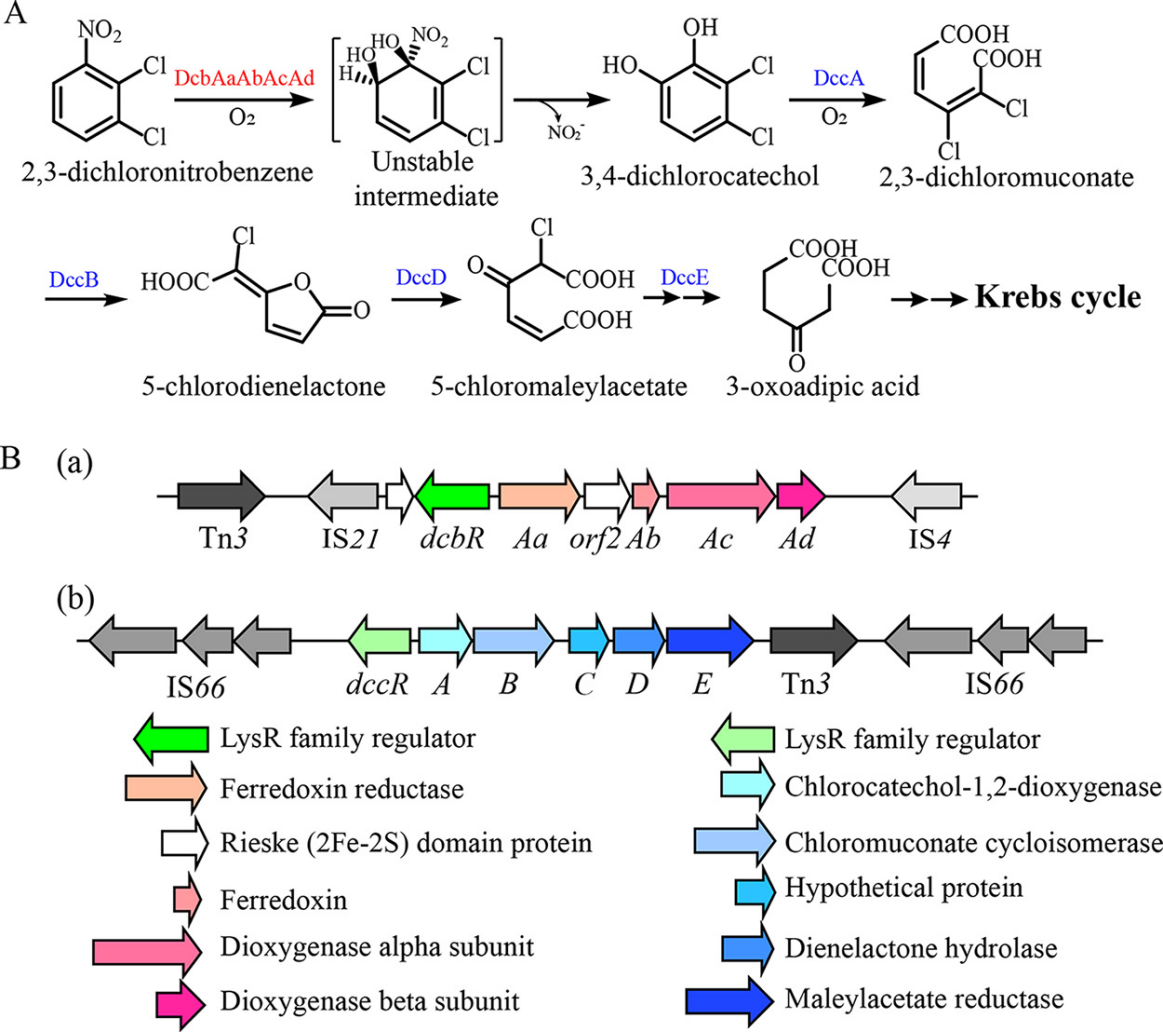
The catabolic pathway of 2,3-dichloronitrobenzene (23DCNB) in *Diaphorobacter* sp. strain JS3051 and candidate genes encoding the enzymes. (A) Proposed pathway of 23DCNB catabolism. (B) Organizations of the *dcb* gene cluster encoding the Nag-like dioxygenase (a) and the *dcc* gene cluster encoding the chlorocatechol catabolic enzymes (b). The identification of *dcb* genes was based on the 2-chloronitrobenzene (2CNB) dioxygenase from Pseudomonas stutzeri ZWLR2-1 and the *dcc* genes were based on the 3- and 4-chlorocatechol catabolic genes (*clc* genes) from Pseudomonas knackmussii B13. The flanking mobile elements are shown as different shades of gray.

A previous study provided preliminary evidence that strain JS3051 degrades 23DCNB by an oxidative pathway (9), similar to the pathway of 2CNB in Pseudomonas sp. strain ZWLR2-1 (20). Therefore, a working hypothesis for an analogous pathway for 23DCNB (Fig. 1A) provided the basis to search for candidate genes in JS3051. First, a gene cluster (designated *dcb*, Fig. 1B) encoding a three-component dioxygenase was a strong candidate for involvement in the initial dihydroxylation of 23DCNB due to its similarity to the ring-hydroxylating dioxygenases responsible for the catabolism of naphthalene and nitroarenes (Table S3). Second, the enzyme catalyzing the ring cleavage in the 23DCNB pathway was likely to be a chlorocatechol or catechol dioxygenase. A gene cluster (designated *dcc*, Fig. 1B) is highly similar to the *clc* genes responsible for 3- and 4-chlorocatechol oxidation in Pseudomonas knackmussii B13 (29). Additionally, two other catechol 1,2-dioxygenase genes were also annotated on the chromosome of strain JS3051. The *dcb* and *dcc* clusters are not contiguous on the chromosome (Fig. 1B). Divergently transcribed LysR family regulators (*dcbR* and *dccR*, respectively) are present on both clusters. Gene annotations, locations on the chromosome, and the most closely related matches are listed in Table S4.

### A Reiske-iron dioxygenase catalyzes the dihydroxylation of 23DCNB to 34DCC.

**FIG 2:**
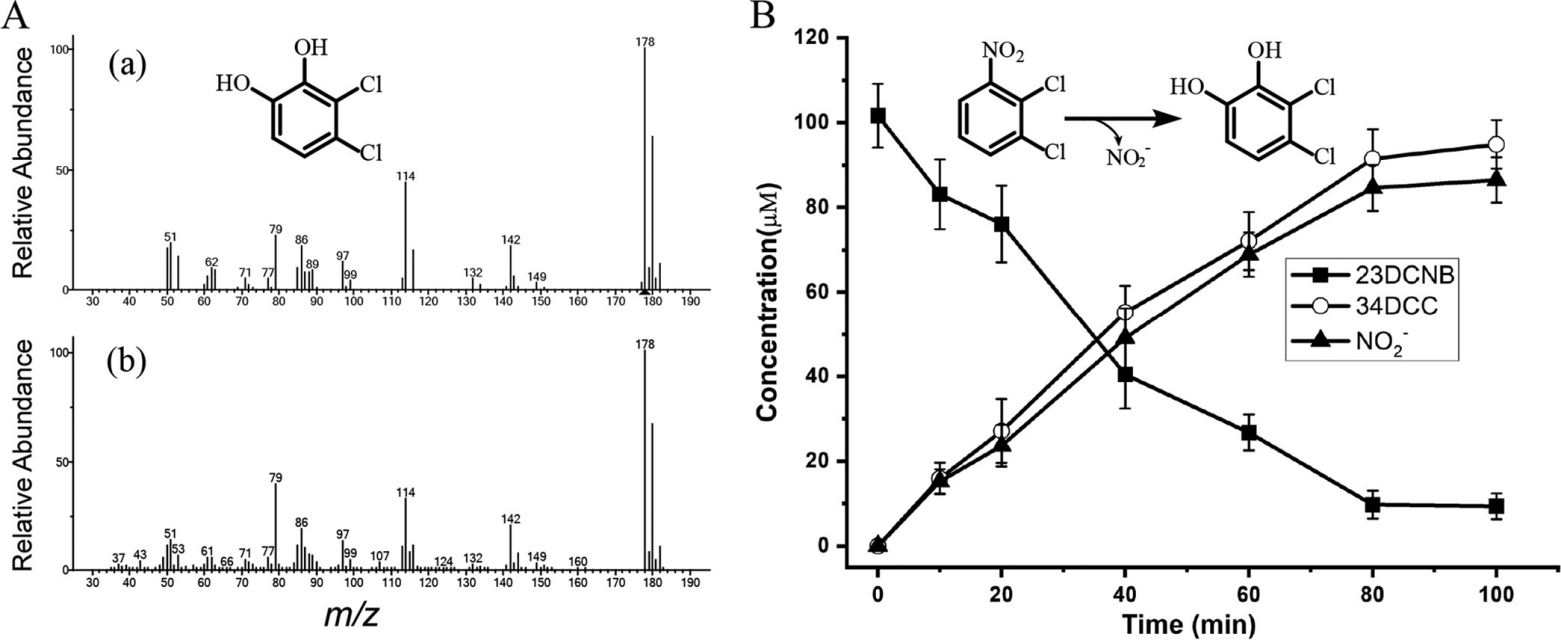
Whole-cell biotransformation of 23DCNB by E. coli cells carrying pETDuet-DCB. (A) GC-MS identification of the product from biotransformation of 23DCNB catalyzed by DcbAaAbAcAd. (a) Mass spectrum of authentic 34DCC; (b) Mass spectrum of the product from the whole-cell biotransformation of 23DCNB by E. coli cells carrying pETDuet-DCB. (B) Time course of 23DCNB biotransformation by E. coli cells harboring pETDuet-DCB. The results shown are average values of three technical replicates of a representative experiment, and all independent experiments had similar results. Error bars indicate standard deviations.

The genes encoding the predicted 23DCNB dioxygenase (*dcbAaAbAcAd*) are related to the Rieske non-heme iron oxygenases, comprising an oxidoreductase (DcbAa), an iron-sulfur ferredoxin protein (DcbAb), and a terminal oxygenase (α-subunit DcbAc and β-subunit DcbAd). To determine whether the putative *dcbAaAbAcAd*-encoded dioxygenase is responsible for the initial dihydroxylation reaction in 23DCNB degradation, *dcbAaAbAcAd* from strain JS3051 was cloned into pETDuet-1 and expressed in Escherichia coli strain BL21(DE3) cells. When a whole-cell biotransformation assay was performed with 23DCNB as the substrate, a single product was detected by high-performance liquid chromatography (HPLC) and identified as 3,4-dichlorocatechol (34DCC) based on comparison of the retention time and UV absorption spectrum with those of authentic 34DCC (Fig. S2). The identity was further confirmed by gas chromatography-mass spectrometry (GC-MS) analysis (Fig. 2A). During the biotransformation, 23DCNB was converted stoichiometrically to 34DCC and NO_2_^−^ (Fig. 2B). The results indicated that DcbAaAbAcAd is a 23DCNB dioxygenase capable of oxidizing 23DCNB to 34DCC with concomitant nitrite release. No other nitroarene dioxygenase candidates were found in the genome of JS3051.

### The amino acid at position 204 changes the substrate specificity of 23DCNB dioxygenase (23DCNBDO) and 2NT dioxygenase (2NTDO).

**FIG 3:**
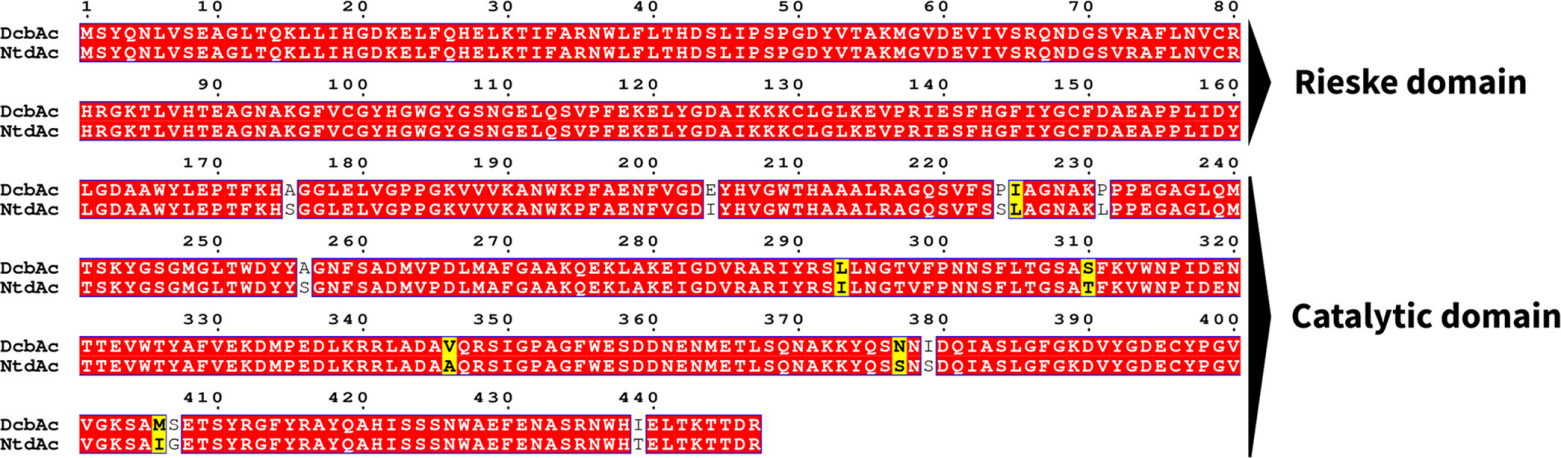
Amino acid sequence alignment of the α subunits of 23DCNB dioxygenase (DcbAc) and 2-nitrotoluene dioxygenase (NtdAc). Identical amino acids are displayed in white font on a red background. Different amino acids are displayed in black font and amino acid mutations sharing similar properties are shown on a yellow background. The Rieske domains, which are involved in electron transfer, are 100% identical. The 14 differing residues are found in the catalytic domain.

**FIG 4:**
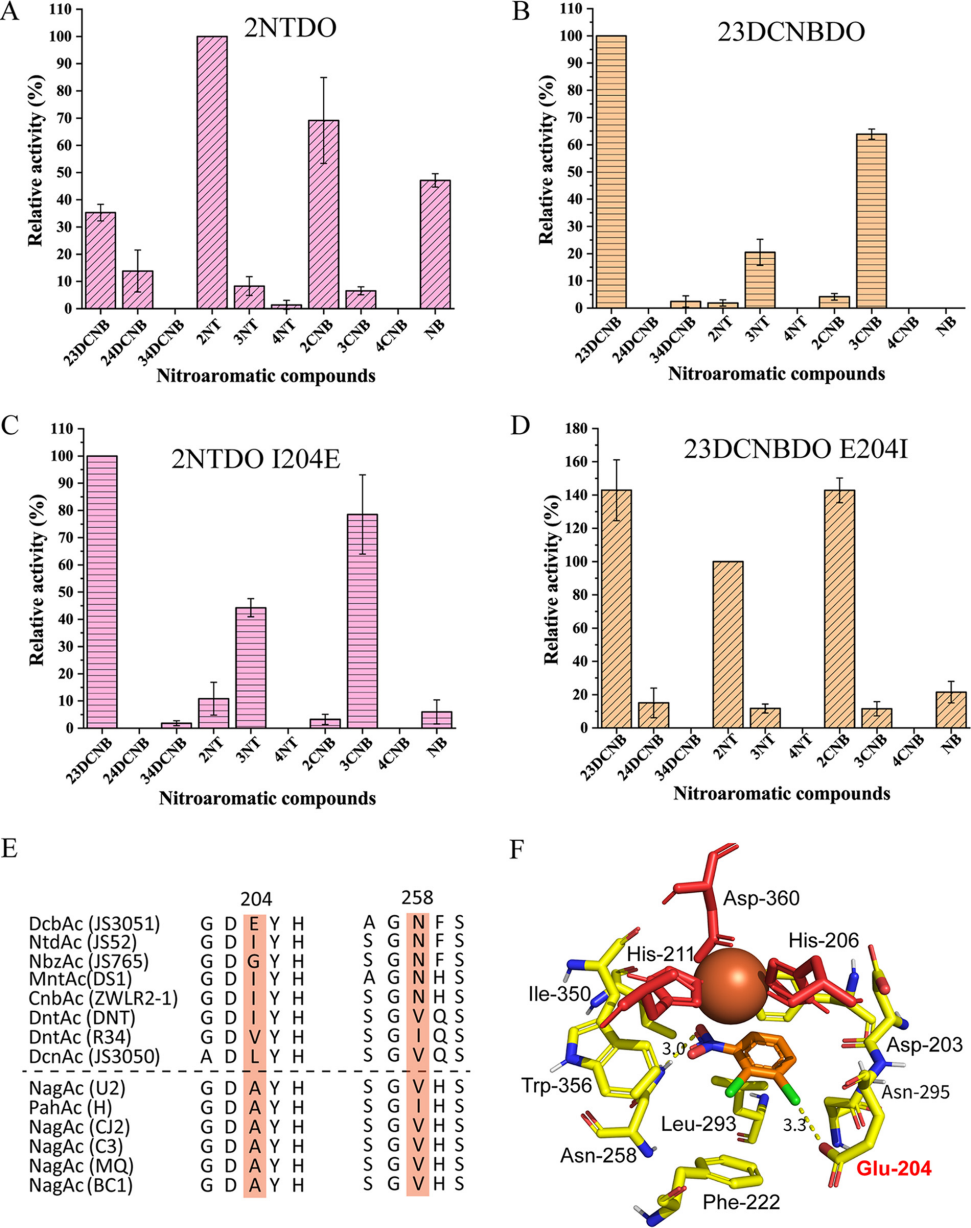
The effect of the amino acid at position 204 on the substrate specificities of 2NTDO and 23DCNBDO. (A to D) Substrate specificity of wild-type 2NTDO (A), 23DCNBDO (B), 2NTDO I204E mutant (C), and 23DCNBDO E204I mutant (D) shown by relative activities monitored with whole-cell nitrite assays in E. coli. For 2NTDO and 23DCNBDO E204I mutants, the relative activities were compared with 2NT (3.1 μmol mg^−1^min^−1^ and 1.1 μmol mg^−1^min^−1^, respectively). For 23DCNB and 2NTDO I204E mutant, the relative activities were compared with 23DCNB (2.6 μmol mg^−1^min^−1^ and 1.5 μmol mg^−1^min^−1^, respectively). The red and brown colors represent the 2NTDO- and 23DCNBDO-derived enzymes, respectively. The diagonal stripe shows the 2NTDO-like activity and the horizontal stripe shows the 23DCNBDO-like activity. The homology model of 23DCNBDO with 23DCNB in the active site was generated to gain insight into the relationship of key residues and substrate specificity. (E) A cropped multiple sequence alignment of representative sequences of α subunit of Nag-like dioxygenases. (F) The residues of the active site are represented in stick format. The mononuclear iron is shown as a sphere (colored brown) and the residues coordinating the mononuclear iron are shown as stick (red). Abbreviations: 23DCNB, 2,3-dichloronitrobenzene; 24DCNB, 2,4-dichloronitrobenzene; 34DCNB, 3,4-dichloronitrobenzene; 2NT, 2-nitrotoluene; 3NT, 3-nitrotoluene; 4NT, 4-nitrotoluene; 2CNB, 2-chloronitrobenzene; 3CNB, 3-dichloronitrobenzene; 4CNB, 4-dichloronitrobenzene; NB, nitrobenzene.

DcbAc, the large subunit determining the substrate specificity, shows highest identity (97%) to its counterpart in 2NT dioxygenase from strain JS42. The enzyme 2NT dioxygenase (2NTDO) was reported to transform chloronitrobenzenes (24), but its ability to transform dichloronitrobenzenes was not reported. All 14 amino acid differences between 23DCNBDO and 2NTDO are located in the catalytic domain near the C-terminus (Fig. 3). In order to investigate the impact of these substitutions on the substrate specificity, relative activities of 23DCNBDO and 2NTDO toward different nitroarenes were analyzed. Although nitrobenzyl alcohols are also sometimes side products from methyl-substituted nitroarenes (30), we focus here on the ring-dihydroxylated products. The enzyme 2NTDO is active not only with 2NT, but also with NB, 2CNB, and 23DCNB (Fig. 4A). In contrast, 23DCNBDO exhibits a preference for *meta*-substituted substrates, such as 23DCNB, 3CNB, and 3NT (Fig. 4B). Both 2NTDO and 23DCNBDO had minimal activity toward *para*-substituted nitroaromatic substrates, including 4NT and 4CNB (Fig. 4A and B).

To determine the contributions of the 14 differing amino acids to substrate specificities of 23DCNBDO and 2NTDO, mutants were made by replacing each of the amino acids present in one protein with the corresponding amino acid of its counterpart. Activity assays indicated that the residue at position 204 plays a key role in determining the specificity of 23DCNBDO and 2NTDO (Fig. 4C and D), whereas the other 13 residues had minimal effect (Table S5). The single replacement of Ile to Glu at position 204 of 2NTDO reduced the activities by 19-, 46-, and 17- fold with 2NT, 2CNB, and NB, respectively. In contrast, the activity with 23DCNB (9-fold higher than that of 2NT) became the primary activity (Fig. 4C). Likewise, the E204I mutation of 23DCNBDO caused a shift in preference from *meta*- to *ortho*-substituted substrates, such as 2CNB, 2NT, and 23DCNB (Fig. 4D). Unexpectedly, 23DCNB was still a primary substrate of the 23DCNBDO E204I variant, indicating that some of the other 13 amino acids affect the activity toward 23DCNB.

### E204 is a unique residue among Nag-like dioxygenases.

Alignment of the amino acid sequences of the α subunits of Nag-like dioxygenases showed that the residues at position 204 (or the corresponding residues) appear to be variable in nitroarene dioxygenases while conserved in naphthalene dioxygenases (Fig. 4E). All of the residues are nonpolar amino acids except for the glutamic acid of 23DCNBDO (Fig. 4E).

To gain insight into how E204 affects the substrate specificity of 23DCNBDO, a homology model of the 23DCNBDO α subunit was constructed based on the crystal structure of NBDO (95% amino acid sequence identity) (31). The 23DCNBDO protein has a similar hydrophobic pocket to that of other Nag-like dioxygenases, with residues including Phe200, Leu293, Leu305, and Phe222 (Fig. 4F). The isoleucine at position 204 of 2NTDO also contributes to the hydrophobic environment in the active site. Substituting the Ile204 with a glutamic acid changes the hydrophobic environment around the C_3_ atom of 2NT and, consequently, affects its correct positioning. On the other hand, the glutamic acid seems to interact with the C_3_ chlorine atom through a halogen bond (Fig. 4F) (32), which could be the cause of the higher activity for 3CNB and 23DCNB than for 3NT (Fig. 4B and C).

### Structural comparison of nitroarene dioxygenases.

**FIG 5:**
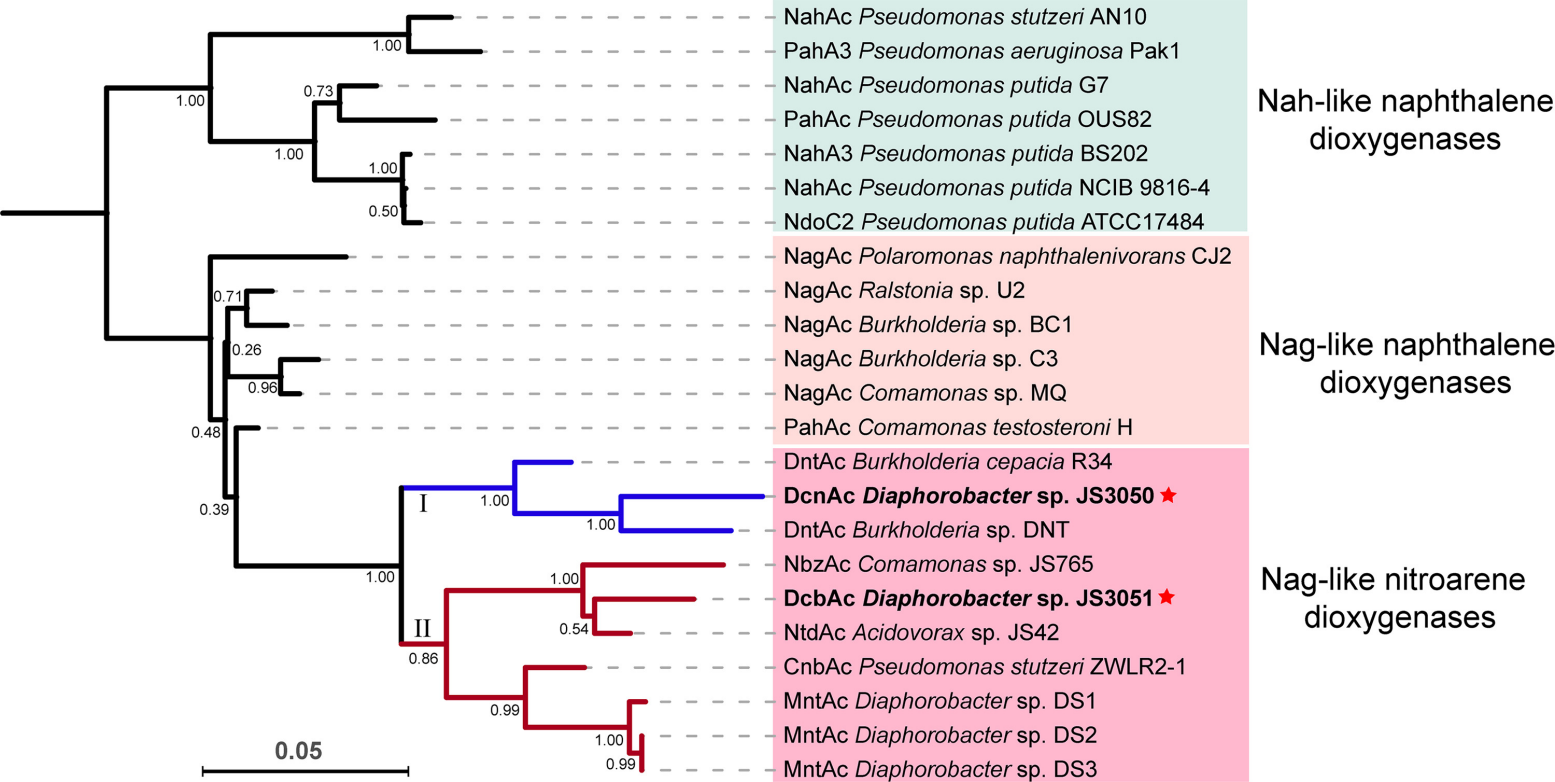
Phylogenetic tree of naphthalene and nitroarene dioxygenases. The tree was constructed based on the amino acid sequences of dioxygenase α subunits and performed by MEGA utilizing the neighbor-joining method with bootstrap replications of 1,000. The numbers at each of the branching nodes indicate bootstrap values. The red stars indicate the 23DCNB and 34DCNB dioxygenases compared in this study.

The lack of activity of both 2NT and 23DCNB dioxygenases toward 34DCNB (Fig. 4A and B) is consistent with the observation that the system in JS3051 is not closely related to that of JS3050 (Fig. 5), which was isolated from the same habitat based on its ability to degrade 34DCNB. Substrate specificity assays revealed that 24DNT dioxygenase accepts 34DCNB, but not 23DCNB, as a substrate for ring hydroxylation (data not shown), which is consistent with the phylogenetic analysis of Nag-like dioxygenases (Fig. 5). The 23DCNB and 34DCNB dioxygenases share more similar substrate preferences with 2NT dioxygenase and 24DNT dioxygenase than with each other.

**FIG 6:**
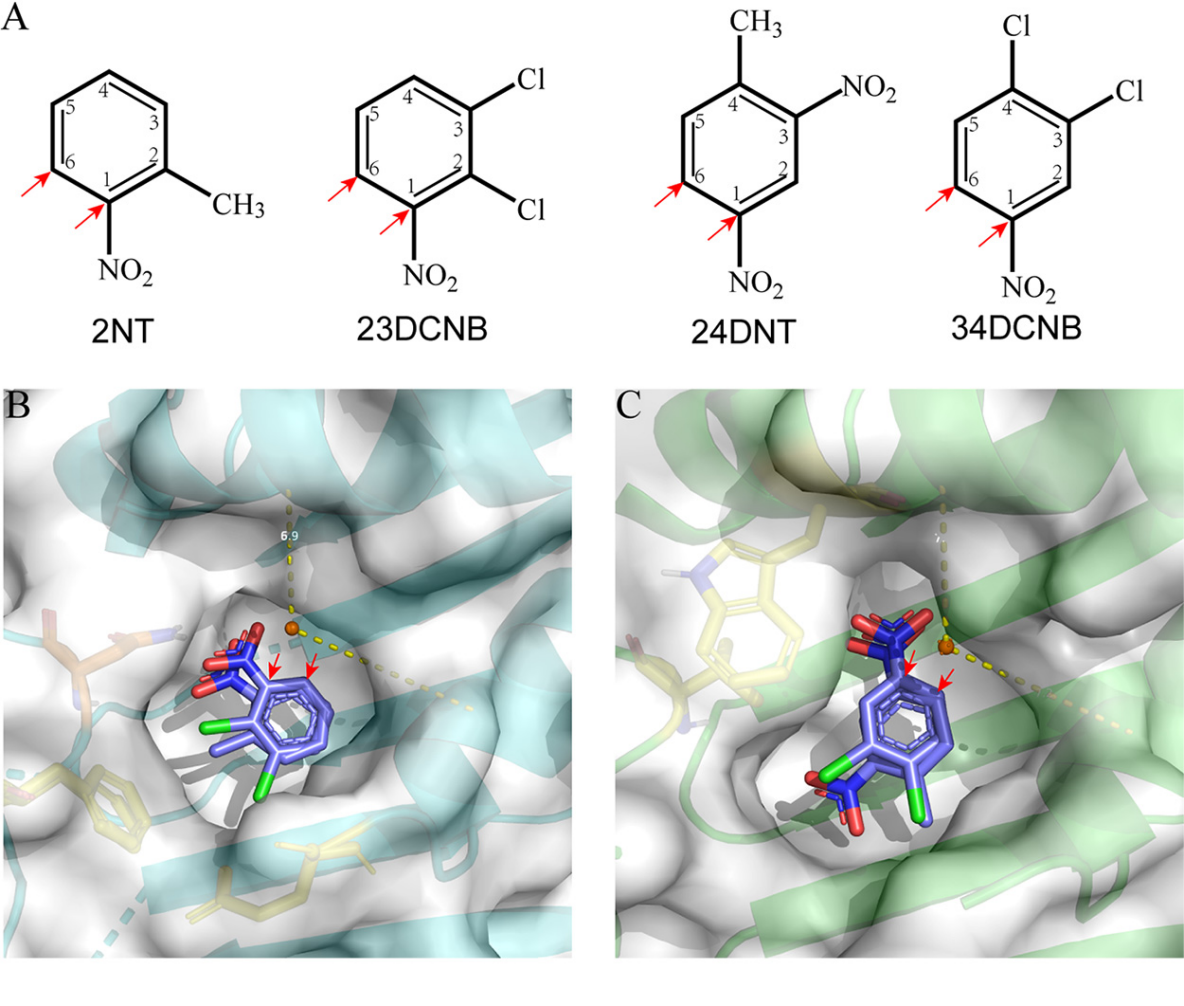
Orientations of nitroaromatic substrates in the active center. (A) The dihydroxylated position of corresponding nitroarenes. (B) Orientation of 2NT and 23DCNB in the substrate-binding pocket of 2NT dioxygenase. (C) Orientation of 24DNT and 34DCNB in the substrate-binding pocket of 24DNT dioxygenase. Red arrows indicate the attack sites of dihydroxylation. Substrates are represented by stick format: chlorine (green), carbon (light blue), nitrogen (dark blue), oxygen (red).

Identification of products revealed that the dihydroxylation occurred at the analogous positions of 2NT and 23DCNB (both have a C_2_ substituted group) and also the analogous positions of 24DNT and 34DCNB (both have C_3_ and C_4_ substituted groups) (Fig. 6A). Molecular docking indicated that 2NT dioxygenase accommodates both 2NT and 23DCNB in similar orientation in the substrate-binding pocket (Fig. 6B). Notably, the pocket shows strong steric hindrance to the C_4_ substituted groups. This is supported by the fact that 2NT dioxygenase has a 6-fold higher activity for 2CNB than for 24DCNB. Similarly, both 2NT and 23DCNB dioxygenases have minimal activity with 4NT and 4CNB (Fig. 4A and B). In the same way, 24DNT and 34DCNB also have similar orientation in the substrate-binding pocket of 24DNT dioxygenase, which accounts for the regiospecific dihydroxylation (Fig. 6C). Residue 258 of the 24DNT dioxygenase α subunit is a valine, and it does not form a hydrogen bond with the nitro group as does the Asn258 in 2NT dioxygenase. In contrast, the Val258 and Trp256 are very close to the C_2_ of the substrates (∼3.6 Å), and thus increase the steric hindrance to the C_2_ substituted group (Fig. 6C).

### DccA catalyzes the ring cleavage of 34DCC.

**FIG 7:**
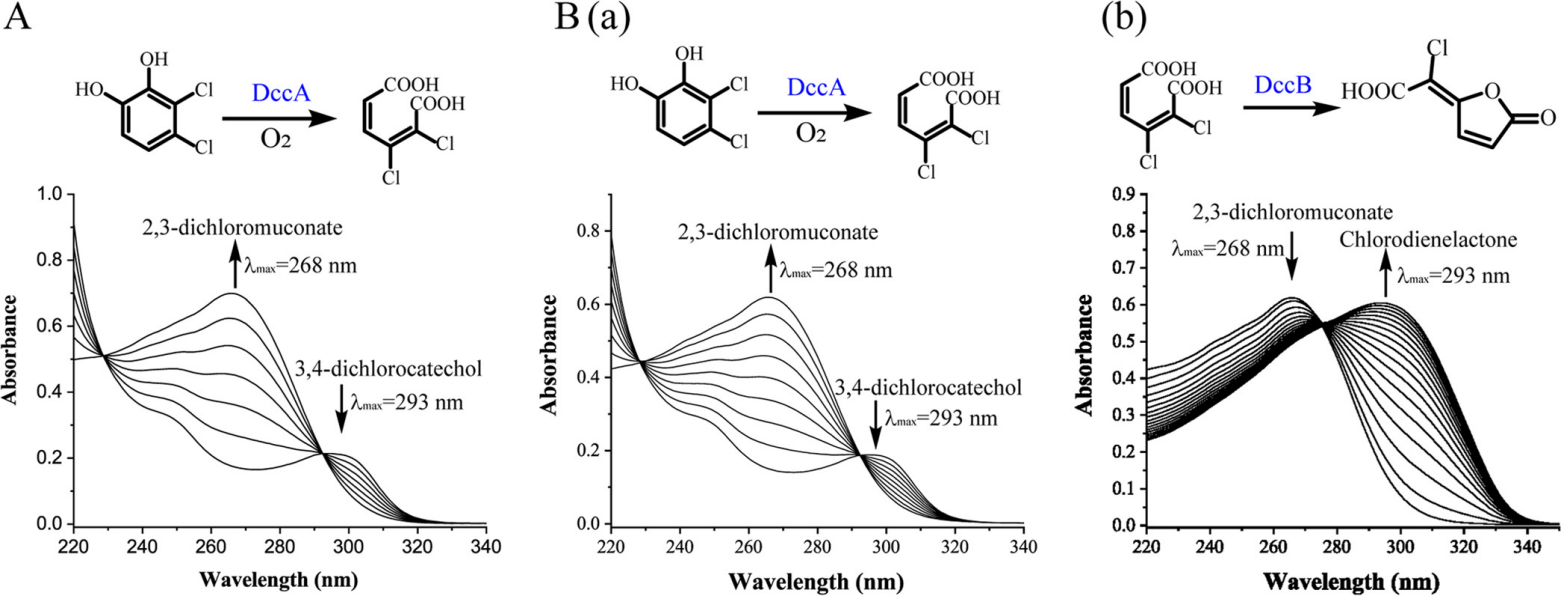
DccA and DccB catalyze ring-cleavage of 34DCC and cycloisomerization of 2,3-dichloromuconate. (A) Activity of DccA toward 34DCC. The reaction mixture contained Tris-HCl buffer (pH 8.0) and 3 μg of crude enzyme prepared from E. coli cells expressing DccA. The reaction was initiated by the addition of 34DCC to a final concentration of 50 μM. (B) Assays of DccA and DccB enzyme activities by sequential catalytic reactions using 34DCC as the starting substrate. The reaction mixture contained Tris-HCl buffer (pH 8.0) and 10 μg of crude enzyme prepared from E. coli cells coexpressing DccA and DccB. The reaction was initiated by the addition of 34DCC to a final concentration of 50 μM. (a) Spectral change during ring cleavage reaction of 34DCC by DccA (scanned every 10 s). (b) Spectral shift during conversion of 2,3-dichloromuconate by DccB (scanned at 1 min intervals).

The downstream genes involved in the catabolism of 34DCC were further investigated. BLAST analysis revealed three candidate genes (*I3K84_08270*, *I3K84_12300*, and *I3K84_15960*) encoding putative (chloro)catechol dioxygenases that might catalyze 34DCC ring cleavage. Among them, only the *I3K84_08270* (designated *dccA*) encoded an enzyme able to catalyze conversion of 34DCC into 2,3-dichloromuconate (Fig. 7A). Cell extract from E. coli carrying DccA exhibited a specific activity of 0.37 U/mg for catechol and 64% relative activity toward 34DCC. Cell extracts from strain JS3051 exhibited a specific activity of 0.08 U/mg for 34DCC and its relative activity against 34DCC (76%) was similar to that from E. coli cells carrying DccA (Table 1). The results are consistent with the hypothesis that DccA catalyzes the 34DCC ring cleavage in strain JS3051. The kinetic parameters of purified H_6_-DccA (Fig. S3) indicate that it has a higher affinity for 34DCC (*K_m_*, 0.48 μM) and 4CC (*K_m_*, 0.73 μM) among the tested substrates (Table 2). H_6_-DccA also had similar catalytic efficiency for 34DCC (*k_cat_*/*K_m_*, 44.2 min^−1^μM^−1^) and 4CC (*k_cat_*/*K_m_*, 53.6 min^−1^μM^−1^), suggesting that DccA has successfully adapted to the 23DCNB pathway (Table 1). Absence of activity toward 45DCC, a 34DCC analogue and the only product from 34DCNB degradation in strain JS3050, indicates that DccA has high substrate specificity toward 34DCC.

**TABLE 1:** Relative catechol dioxygenase activities of cell extracts from E. coli cells carrying DccA or ClcA and strain JS3051 grown on 23DCNB

Substrate	% Relative activity^*a*^ of cell extract of E. coli DccA (U/mg)	% Relative activity of cell extract of strain JS3051 (U/mg)	% Relative activity of cell extract of E. coli ClcA^*b*^ (U/mg)
Catechol	100	100	100
3MCC	198.6	116.9	299.3
4MCC	229.5	278.3	253.7
3CC	85.4	106.0	137.1
4CC	128.4	177.1	94.9
34DCC	64.3	75.9	3.4
45DCC	1.6	ND^*c*^	ND^*c*^

a Relative activity is expressed as the percentage of the specific activity compared with catechol set as 100%. The specific activities of DccA extract, JS3051 extract, and ClcA extract toward catechol are 0.37 ± 0.02, 0.08 ± 0.01, and 0.29 ± 0.02, respectively. Assays were done in triplicate and standard deviation among replicates was less than ±5%.

b ClcA was from Pseudomonas knackmussii strain B13.

c ND, not detected.

**TABLE 2:** Kinetic parameters of H_6_-DccA for catechol and substituted catechols

Substrate	*K*_m_ (μM)	*V*_max_ (μM/min)	*K*_cat_ (min^−1^)	*K*_cat_/*K*_m_ (min^−1^ μM^−1^)
Catechol	9.06 ± 0.88	0.44 ± 0.01	21.8	2.4
3MC	12.85 ± 1.52	0.63 ± 0.03	31.5	2.5
4MC	10.40 ± 1.35	4.63 ± 0.27	231.5	22.3
3CC	2.58 ± 0.38	0.42 ± 0.02	21.0	8.1
4CC	0.73 ± 0.06	0.78 ± 0.01	39.2	53.6
34DCC	0.48 ± 0.10	0.43 ± 0.02	21.3	44.2
45DCC	ND^*a*^	ND	ND	ND

a ND, not detected.

### DccB catalyzes conversion of 2,3-dichloromuconate to chlorodienelactone.

DccB has 100% sequence identity to the well-studied chloromuconate cycloisomerase ClcB_B13_ that catalyzes lactonization of 2,4-dichloromuconate, 2-chloromuconate, and 3-chloromuconate (33). However, its activity toward 2,3-dichloromuconate, the ring-cleavage product of 34DCC, was unknown. Heterologously coexpressed DccA and DccB were used in an *in vitro* sequential catalytic assay to detect the cycloisomerization of 2,3-dichloromuconate with 34DCC as the initial substrate. The reaction catalyzed by DccA was completed rapidly and, due to the relatively slow reaction rate of DccB, the 2,3-dichloromuconate (λ_max_ = 268 nm) accumulated in the reaction mixture (Fig. 7B), followed by a slower spectral shift from 268 nm to 293 nm (Fig. 7C), consistent with formation of 5-chlorodienelactone (33). The isobestic point at 275 nm indicated direct conversion. The spectral change was not detected with the crude extracts containing DccA only, indicating that DccB is a functional chloromuconate cycloisomerase acting on 2,3-dichloromuconate as substrate.

## DISCUSSION

This study revealed that the catabolism of 23DCNB by strain JS3051 is initiated by a Rieske-type 23DCNB dioxygenase that adds both atoms of molecular oxygen to the benzene ring with the release of nitrite and formation of 34DCC. The 34DCC product is subsequently degraded via a modified *ortho*-cleavage pathway (Fig. 1A). The evolution of the 23DCNB pathway is most likely from an ancestral Nag-like naphthalene degradation pathway and a chlorocatechol pathway, both with modified enzyme specificity. Analyses of the 23DCNB dioxygenase using biochemical and structural approaches also revealed key factors determining substrate specificity and the recent divergence of nitroarene dioxygenases.

### The 23DCNB dioxygenase genes (*dcb*) share a recent common ancestor with the genes encoding 2-nitrotoluene dioxygenase.

**FIG 8:**
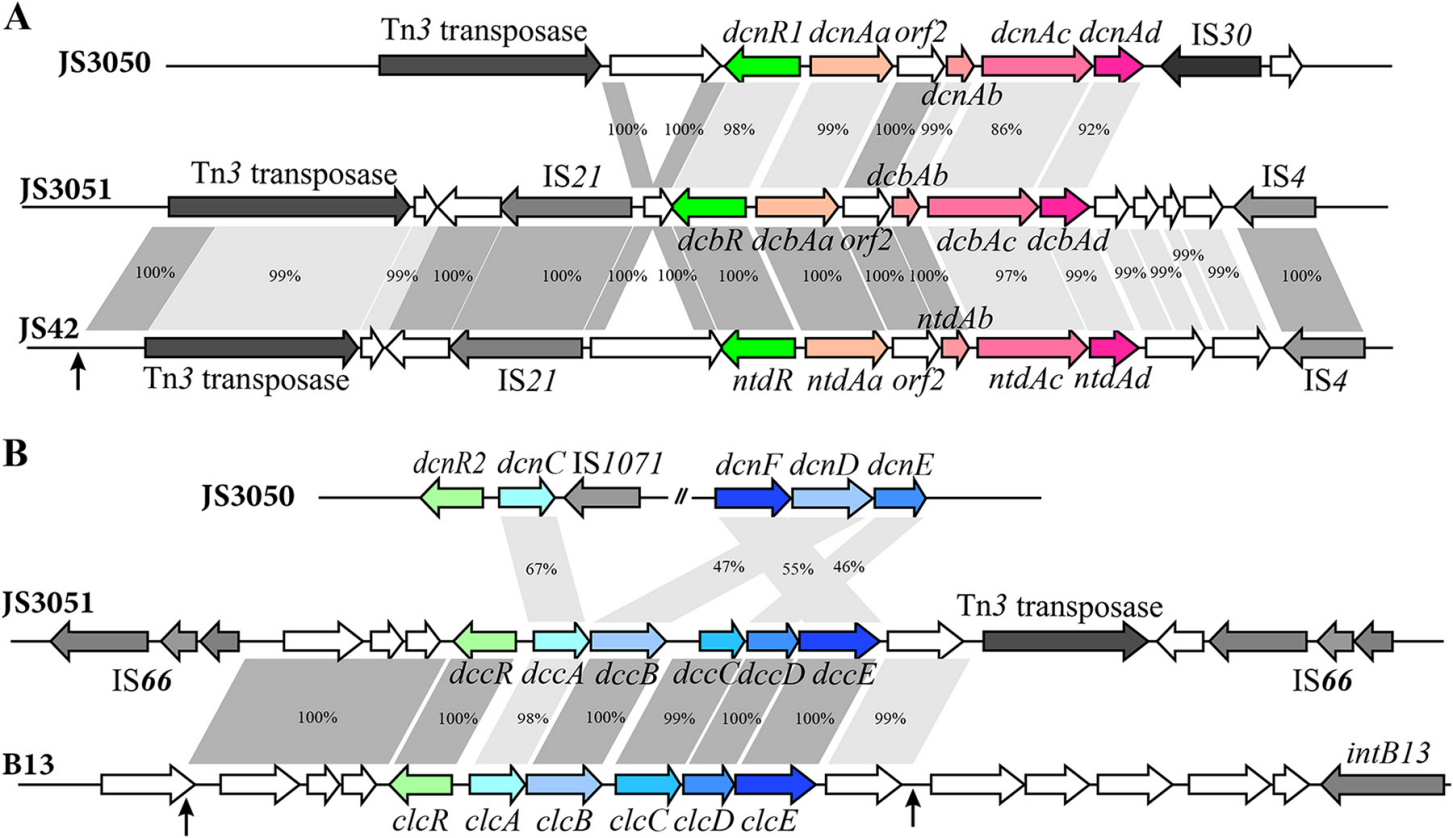
The 23DCNB catabolic pathway assembled from two origins. (A) Comparison of the *dcb* gene cluster with its counterparts from strain JS42 and strain JS3051, as well as their flanking mobile elements. (B) Comparison of the *dcc* gene cluster with the counterparts from strain JS3050 and the *clc* element from strain B13. The black arrows indicate the boundaries of homologous fragments.

The conclusion that the *dcb* genes share a recent common ancestor with the genes encoding 2-nitrotoluene dioxygenase is well supported by the surprisingly high identity and the same organization between *dcb* genes of strain JS3051 and the *ntd* genes from the 2-nitrotoluene degrader strain JS42 (Table S3) (Fig. 8A). Additional evidence dictated that *orf2* within the *dcb* operon has the same start codon and high similarity with the N terminus of salicylate hydroxylase large subunits (NagG) from strains containing *nag*-like genes, such as strain U2. The truncated *nagG* remnant is a strong indication that *dcb* originated from a *nag*-like naphthalene dioxygenase gene cluster (14). The identical sequence between *orf2* and its counterpart in the *ntd* operon (34) (Fig. 8A) indicates the close evolutionary relationship of strains JS3051 and JS42.

Transposable elements are often responsible for transfer of catabolic genes during adaptive evolution in response to the introduction of xenobiotics (35–37). The *dcb* cluster is surrounded by a single copy of an IS*21-*like insertion sequence, together with a Tn*3* transposase gene upstream and an IS*4* family insertion sequence downstream, that are identical to those in the *ntd* cluster from the 2NT degrader *Acidovorax* sp. strain JS42 (Fig. 8A). Additionally, a TnpA transposase, an IS*91*, an IS*Csp2*, and two copies of IS*1071* transposons are flanked by the upstream Tn*3* transposase in JS3051 (Fig. S4). All are absent from the *ntd* cluster but located on other sites of the chromosome or the plasmid pAOVO01 of strain JS42. The above evidence, together with the close phylogenetic relationship between strains JS3051 and JS42 (Table S2), suggests strongly that the *dcb* cluster originated from a within-species lineage related to strain JS42.

### Structure-activity relationships among nitroarene dioxygenases.

The striking similarity among the large subunits provides strong support for the previous observation that all the genes encoding Nag-like nitroarene dioxygenases (Fig. 5) share a recent common ancestor with the Nag-like naphthalene dioxygenases (18). The divergence of the sequences, substrate specificities, and structural characteristics of nitroarene dioxygenases can be summarized in the following features. The 24DNT and 34DCNB dioxygenases are in a clade separated from the other known nitroarene dioxygenases (Fig. 5). The clade II nitroarene dioxygenases accept 2/3-substituted nitroarenes, whereas the clade I nitroarene dioxygenases prefer 3,4-substituted substrates (Fig. 4A and B) (21, 31, 38–40). The position of the substituents seems to have more influence than the type of the groups on the substrate preferences of nitroarene dioxygenases. The catalytic domains of nitroarene dioxygenases share many common features, but notable divergence is introduced by residue 258. In class II nitroarene dioxygenases, Asn258 plays an important role in positioning the substrates by interacting with the nitro groups through hydrogen bonding (Fig. 4F) (31). In contrast, the class I nitroarene dioxygenases possess a nonpolar residue (Val or Ile) at position 258, which is consistent with Nag-like naphthalene dioxygenases (Fig. 4E). Other conserved residues in the catalytic domain, including I207, A223, A254, Q259, Q293, G309, F314, I329, and D384, are also only present in class I dioxygenases.

Multiple structural features can affect accommodation of new substrates by Reiske dioxygenases. For instance, the channel of the active site of some Reiske dioxygenases can be a bottleneck that controls substrate access (41, 42). Similarly, evolution of 2NT dioxygenase into a productive 4NT dioxygenase resulted from artificial laboratory evolution involving direct selection of spontaneous mutants (15). The increased 4NT activity was attributed to three missense mutations outside the substrate-binding site of the catalytic domain. In contrast, the residue 204, which determines the substrate specificities of 2NTDO and 23DCNBDO observed in this study, is located in the substrate-binding site (Fig. 4F). Understanding the mechanisms of substrate selectivity of nitroarene dioxygenases will be beneficial for the potential applications in creating variants with novel activities.

### A chlorocatechol cluster involved in the lower pathway of 23DCNB catabolism.

The evolution of new substrate preferences that allowed the initial attack and elimination of the nitro groups of the various compounds was necessary but not sufficient for assembly of productive catabolic pathways. In the downstream pathway of 23DCNB catabolism, the striking similarity between the entire *dcc* and *clc* clusters, the presence of the almost identical flanking fragments between these two clusters, and the large fragment containing the *dcc* cluster flanked by two direct repeats of IS*66*-family insertion sequences and a Tn*3*-like element (Fig. 8B) strongly indicate that the *dcc* genes were recruited through recent lateral transfer. The facts that the positions of *ntd* and *cat* in JS42 are similar to those of *tcb* and *cat* in the genome of JS3051 and the *dcc* genes are absent from the genome of JS42 support the argument that the *dcc* genes of JS3051 were recruited by horizontal gene transfer.

In addition to recruitment, the subsequent evolution of genes encoding enzymes with modified specificities for the downstream pathways would have been essential. Although ClcA_B13_ and DccA differed by only six amino acids (Table S6), ClcA_B13_ exhibited a strong preference for 35DCC (43) rather than 34DCC (Table 1). This situation is similar to that of CbnA (preferring 35DCC) and TcbC (preferring 34DCC), which differ in 12 amino acids (44). Sequence analysis indicates that both DccA and TcbC share the same Val48, Ala52, and Met73 residues, and both CbnA and ClcA_B13_ share the same Leu48, Val52, and Ile73 residues (Table S6). These three residues seem to be responsible for the preferential activity toward 34DCC and 35DCC (44).

### Different recent origins of genes encoding catabolic pathways for DCNB isomers in strains JS3050 and JS3051.

Although the two closely related strains isolated from the same location utilize Nag-like nitroarene dioxygenases to catalyze the initial dioxygenation reaction of DCNBs, several lines of evidence indicate their different recent ancestries. First, the 34DCNB dioxygenase is closer to 24DNT dioxygenase than to 23DCNB dioxygenase in sequence, structure, and substrate specificity. Second, an IS*30*-like insertion sequence flanked by the *dcnAd* of JS3050 is identical to that of strain DNT (45), and totally different from the mobile elements of JS3051 in organization and sequence (Fig. 8A). Finally, the genes involved in the chlorocatechol pathway were discontinuously distributed on the chromosome and a plasmid of strain JS3050 and showed relatively low identity with the contiguous *dcc* genes in strain JS3051 (Fig. 8B). Analyses of substrate compatibility in the active sites (Fig. 6), combined with biochemical characterization, provided insight into the molecular mechanisms underlying the different origins of DCNBs dioxygenases. It is clear that the active sites of the different precursor dioxygenases evolved separately for their respective DCNB substrates. The lower pathways seem to have been selected by the regiospecific differences in metabolites of DCNBs. Theoretically, the dioxygenation of 34DCNB could generate both 34DCC and 45DCC. In fact, 34DCNB dioxygenase from JS3050 specifically transformed 34DCNB to 45DCC (21). Such regiospecificity would be consistent with the presence of a 45DCC pathway in JS3050, whereas a 34DCC pathway is required for productive degradation of 2,3DCNB by JS3051.

## MATERIALS AND METHODS

### Chemicals, bacterial strains, and culture conditions.

All chemicals were purchased from Sigma-Aldrich with the following exceptions: 34DCNB, 23DCNB, and 2NT (Macklin, China), 3,4-dichlorocatechol (CFW Laboratories Inc, USA), and 3-chlorocatechol (TCI, Japan). Bacterial strains and plasmids used in this study are listed in Table 3. *Diaphorobacter* sp. strain JS3051 was grown at 30°C in half-strength mineral salts broth (MSB) (46), pH 7.0, supplemented with 23DCNB (1 mM) and Amberlite XAD-7 resin (Sigma-Aldrich) (3.5 g/liter). E. coli strains DH5α and BL21(DE3) used for cloning and expressing recombinant proteins, respectively, were cultured at 37°C in lysogeny broth (LB) or LB agar with appropriate antibiotics (40 μg/ml kanamycin or 100 μg/ml ampicillin).

**TABLE 3:** Strains and plasmids used in this study

Strain or plasmid	Description	Source
Strains		
*Diaphorobacter* sp. strain JS3051	2,3-dichloronitrobenzene degrader	(9)
E. coli DH5α	*supE44 lacU169* (ϕ80d*lacZ* ΔM15) *recA1 endA1 hsdR17 thi-1 gyrA96 relA1*	Novagen
E. coli BL21(DE3)	F^−^ *ompT hsdS_B_*(r_B_− m_B_−) *gal dcm lacY1* (DE3)	Novagen
Plasmids		
pET28-28a(+)	IPTG inducible expression vector, Kan^r^	Novagen
pETDuet-1	IPTG inducible co-expression vector, Amp^r^	Novagen
pET-*dccA*	*dccA* fragment inserted into pET-28a(+) between NdeI and BamHI; Kana^r^	This study
pET-*dccAB*	*dccAB* fragment inserted into pET-28a(+) between NdeI and BamHI; Kana^r^	This study
pET-*clcA*	*clcA_B13_* fragment inserted into pET-28a(+) between NdeI and BamHI; Kana^r^	This study
pETDuet-DCB	NcoI-SacI fragment containing *dcbAaAb* and NdeI-KpnI fragment containing *dcbAcAd* inserted into pETDuet-1; Amp^r^	This study
pETDuet-NTD	NcoI-SacI fragment containing *ntdAaAb* and NdeI-KpnI fragment containing *ntdAcAd* inserted into pETDuet-1; Amp^r^	This study
pETDuet-DCB204	pETDuet-DCB containing the DcbAc-E204I mutation; Amp^r^	This study
pETDuet-NTD204	pETDuet-NTD containing the NtdAc-I204E mutation; Amp^r^	This study

### Genome sequencing and analysis.

*S*equencing of genomic DNA of strain JS3051 was performed by Shanghai OE Biotech Co., Ltd. (Shanghai, China) using the Pacific Bioscience (PacBio) RS technology (47). The complete genome sequence of strain JS3051 was assembled using Falcon (48) and Circulator (49). The genome was annotated by the Prokaryotic Dynamic Programming Genefinding Algorithm (Prodigal V2.6.3) (50) and RAST annotation service (51).

### Site-directed mutagenesis.

Site-directed mutagenesis of *dcbAc* and *ntdAc* was performed by PCR. Briefly, plasmids pETDuet-DCB and pETDuet-NTD were used as templates for mutagenesis. The templates were amplified by *PFU* DNA polymerase (Vazyme Biotech Co., Ltd) following the manufacturer’s protocol with the mutagenic oligonucleotides listed in Table 4. The products were transformed into E. coli DH5α and screened on LB agar with ampicillin.

**TABLE 4:** Oligonucleotides used in this work

Oligonucleotide	Sequence (5′–3′)	Description
DcbAaAb-F	ATGGAACTGGTAGTAGAACCCCT	pETDuet-DCB
DcbAaAb-R	TTAGTCCAGCTTGAGCATCACGCGC	
DcbAcAd-F	ATGAGTTACCAAAACTTAGTGA	
DcbAcAd-R	TCACAGGAAGACCAACAGGTTGT	
DccA-F	ATGGATAAACGAGTTGCCGAGG	pET-*dccA*
DccA-R	TCATGCCACTGTCTCCGTAGC	
DccAB -F	ATGGATAAACGAGTTGCCGAGG	pET-*dccAB*
DccAB -R	TCAACCCGCGCGGGTGAA	
DCBI204E-F	GGAAAACTTTGTAGGTGACATATACCACGTTGGTTGGACG	pETDuet-DCB204
DCBI204E-R	CGTCCAACCAACGTGGTATATGTCACCTACAAAGTTTTCC	
NTDI204E-F	AAACCGTTTGCAGAAAATTTTGTGGGTGATGAGTACCATGTGGGTTGGAC	pETDuet-NTD204
NTDI204E-R	TTTGGCAAACGTCTTTTAAAACACCCACTACTCATGGTACACCCAACCTG	

### Whole-cell biotransformation assays.

To determine the function of *dcbAaAbAcAd*, E. coli strain BL21(DE3) (pETDuet-DCB) was grown in LB medium to an optical density at 600 nm (OD_600_) of 0.6 and gene expression was induced at 30°C for 5 h after addition of IPTG (isopropyl-β-D-thiogalactopyranoside) (0.3 mM). The cells were harvested, washed twice with phosphate-buffered saline (PBS) and suspended in MSB containing 0.1 mM 23DCNB. E. coli strain BL21(DE3) containing the pETDuet-1 vector was used as negative control. Cell suspensions were incubated with shaking (220 rpm, 30°C) and sampled at appropriate intervals for the subsequent analyses. Concentrations of 23DCNB and 34DCC were quantified by HPLC. Nitrite was detected by the Griess method as described previously (52).

### Protein expression and purification.

The *dccA* gene was amplified with primers DccA-F and DccA-R (Table 4) from genomic DNA of strain JS3051 and ligated into expression vector pET-28a(+) between the NdeI and BamHI restriction sites. Recombinant DccA containing an N-terminal 6×His tag was expressed and purified as described previously (53). The purified DccA was used to determine the kinetic parameters toward catechol and substituted catechols. DccA and DccB were coexpressed in E. coli. The *dccAB* sequence was amplified with primers DccAB-F and DccAB-R (Table 4) and ligated to a pET28a(+) vector between NdeI and BamHI sites. Expression conditions and preparation of cell extract containing DccAB were as described above for DccA.

### Enzyme assays and kinetic measurements.

To elucidate the reaction catalyzed by DccA, cell lysates containing DccA were centrifuged at 15,000 × *g* for 60 min to remove the debris. The supernatant was collected and used for enzyme assays. Cell extracts containing ClcA_B13_ and DccAB were prepared the same as for DccA. E. coli BL21(DE3) cells harboring the pET-28a(+) vector were used as a negative control. Extracts were prepared from 23DCNB-grown cells of strain JS3051 by the same method.

The reaction mixture contained crude enzyme (3 to 10 μg protein) in 50 mM Tris-HCl buffer (pH 8.0) and the reaction was initiated by the addition of (chloro)catechol substrates (50 μM). All assays were performed with a Lambda 25 spectrophotometer (PerkinElmer/Cetus, Norwalk, CT). The activities toward catechol or substituted catechols were determined by the increase in absorption at A_260nm_ due to the accumulation of muconate or corresponding chloromuconates. One unit of enzyme activity (U) is defined as the amount of the enzyme required for the production of one μmol of product per min at 25°C. Specific activity is expressed as units per milligram of protein. Purified DccA was used to determine the kinetic parameters as described by Potrawfke et al. (54). The extinction coefficients for chloromuconates reported by Dorn and Knackmuss (55) and Gao et al. (21) were used for determining the 1,2-chlorocatechol dioxygenase activity of DccA. The kinetic curves are shown in Fig. S4.

### Activities of ring-hydroxylating dioxygenases.

The activities of 23DCNBDO, 2NTDO, and their mutants toward different nitroarenes were determined based on the whole-cell biotransformation assay described above with some modifications. Specific activities were obtained by measuring the rates of nitrite accumulation at appropriate intervals (depending on the activity of each dioxygenase). The cells were collected by centrifugation, suspended in equal volumes of 0.1 M NaOH, and boiled for 10 min. Protein concentrations were determined by the Bradford method (56) with bovine serum albumin as the standard.

### Homology model.

Homology models of the α subunit of nitroarene dioxygenases were generated by SWISS-MODEL (57), using the NB dioxygenase α subunit (Protein Data Bank entry: 2BMO) as the template. The sequence identities between nitroarene dioxygenases and the template were more than 85%. The quality of models was estimated based on the QMQE (0.9 to 0.99) and QMEAN (−0.45 to 0) scoring functions. Nitroarene substrates were docked into the active sites of dioxygenase models by AutoDock Vina (58) with default settings. The docking scores for the various poses are shown in Table S7 and the representation of all the different poses are shown in Fig. S6. The productive poses were determined based on the reference structure (31) and docking scores. The structure models were visualized by PyMOL v2.4 (http://www.pymol.org).

### Analytical methods.

Reverse phase high-performance liquid chromatography (HPLC) analyses were carried out with a Waters e2695 separation module equipped with a Waters 2998 photo diode array detector, using a C_18_ reversed-phase column (5 μm, 4.6 × 250 mm) at 30°C. The mobile phases were water containing 0.1% (vol/vol) acetic acid (A) and methanol (B). The elution profile was 20% of solvent B for 5 min, then linear increase to 90% B over 30 min. Gas chromatography-mass spectrometry (GC-MS) analyses were performed with a TRACE 1310 gas chromatograph (Thermo Fisher Scientific Inc., MA, USA) equipped with a capillary column HP-5MS (0.25 mm × 30 m, Agilent technologies., CA, USA). For GC-MS analysis, biotransformation samples were extracted with diethyl ether. Then the extracts were evaporated to dryness and dissolved in anhydrous ethyl acetate.

### Data availability.

The whole-genome sequencing data were deposited in the NCBI database under BioProject identifier (ID) PRJNA680215.
